# The AAHKS Surgical Techniques and Technologies Award: Synovial Fluid Metal Ion Levels as a Biomarker for Aseptic Loosening Following Cemented Total Knee Arthroplasty: A Prospective Study

**DOI:** 10.1016/j.arth.2025.04.067

**Published:** 2025-05-06

**Authors:** Aleksander P. Mika, Courtney E. Baker, Jacob M. Wilson, Jaquelyn S. Pennings, Stephen M. Engstrom, Gregory G. Polkowski, J. Ryan Martin

**Affiliations:** Department of Orthopedic Surgery, Vanderbilt University Medical Center, Nashville, Tennessee

**Keywords:** aseptic loosening, revision TKA, synovial fluid analysis, Total knee arthroplasty, complications, synovial testing

## Abstract

**Background:**

Implant loosening following primary total knee arthroplasty (TKA) remains the most common aseptic indication for revision. Aseptic loosening can be difficult to diagnose, as radiographic findings may be absent or difficult to detect or interpret. Commercially available bone cement contains either barium or zirconium to allow for radiographic detection. We hypothesized that implant loosening leads to abrasion-related release of detectable amounts of metal ions from the implant or bone cement, which may serve as biomarkers of aseptic loosening.

**Methods:**

We prospectively enrolled 50 patients (mean age: 65 years, 52% were women) undergoing revision of a cemented TKA requiring component explantation. Synovial fluid was obtained prior to arthrotomy during revision surgery and analyzed for concentrations of barium, zirconium, titanium, cobalt, and chromium in parts per billion (ppb). The diagnostic utility of each ion for detecting loosening was assessed. The operative surgeon determined whether the femoral and tibial components were well-fixed or loose at the time of removal.

**Results:**

There were 25 (50%) patients who had intraoperatively confirmed component loosening. Patients who had confirmed component loosening had elevated levels of zirconium (median levels: 27 versus 6 ppb, *P* = 0.016) and cobalt (median levels: 16 versus 1.5 ppb, *P* = 0.002) compared to patients who did not have loosening. The most accurate synovial metal ion levels for diagnosing aseptic loosening were cobalt (area under the curve: 0.76 (95% confidence interval = 0.611 to 0.92); *P* = 0.001) and zirconium (area under the curve: 0.79 (95% confidence interval = 0.6 to 0.99); *P* = 0.003). In this series, barium, titanium, and chromium levels were not predictive of component loosening.

**Conclusions:**

Aseptic loosening remains a challenging diagnosis without an available confirmatory test. Although the current sample size is limited, our results indicate that synovial fluid cobalt and zirconium levels are valuable indicators of component loosening. In the absence of definitive X-ray findings, synovial fluid analysis offers a promising diagnostic modality. This test can potentially improve patient outcomes by enabling earlier and more accurate detection of aseptic loosening following primary cemented TKA.

Aseptic loosening is one of the most common long-term failure modalities following total knee arthroplasty (TKA) [1–3]. Data from joint revision centers have demonstrated that aseptic loosening following primary TKA accounts for up to 30% of revisions [3]. Even with the introduction of highly cross-linked polyethylene, aseptic loosening remains a frequent cause of failure [4].

Although massive osteolysis with component migration is easy to diagnose radiographically, component debonding is often associated with only subtle radiographic findings [5]. Identifying aseptic loosening following primary TKA remains a challenging clinical problem [6–8]. The current gold standard for the detection of loosening remains serial radiographs demonstrating implant migration, progressive radiolucencies, or cement mantle fracture. However, the best method to make this diagnosis in the absence of the above findings remains a point of debate. Some authors have advocated for alternative imaging modalities, including bone scans, computed tomography (CT) scans, and single photon emission CT scans [9–11]. However, these imaging modalities all have limitations and are often difficult to interpret. Therefore, better screening and confirmatory tests are needed.

Synovial metal ion levels, particularly cobalt and chromium, are well-established markers for diagnosing metallosis and modular junctional failure in the appropriate clinical context [12,13]. Abrasive wear from loose or debonded TKA implants could potentially lead to the release of cement radiotracers, such as barium or zirconium, as well as implant metal ions, including cobalt, chromium, and titanium. We hypothesized that loose TKA implants release detectable levels of these ions, resulting in higher concentrations in patients who have loosening compared to those who have well-fixed implants. To test this hypothesis, we designed a prospective proof-of-concept study to determine whether there are measurable increases in synovial fluid concentrations of barium, zirconium, titanium, chromium, or cobalt in cases of confirmed component loosening.

## Materials and Methods

Institutional review board approval was obtained before commencing this prospective study. A consecutive series of 50 patients undergoing revision TKA of cemented primary components for any indication was enrolled. Patients who did not have cemented components, patients who had revision components, and those undergoing second-stage reimplantation were excluded.

### Synovial Fluid Collection

All patients underwent revision utilizing the patients’ prior TKA incision. The skin incision was taken down to the extensor mechanism, and medial and lateral soft tissue flaps were developed. Before the arthrotomy, an 18-gauge needle with a 10-mL syringe was used to collect synovial fluid for analysis. The samples were then stored at room temperature until the testing was performed. In most patients (29 of 50), a cement sample was also removed and analyzed by mass spectrometry to determine the most likely radiotracer present in the bone cement. Components were assessed intraoperatively and confirmed to be well-fixed or loose by a fellowship-trained arthroplasty surgeon. Components were considered loose if they could be removed without osteotomes or saws or did not require other methods to disrupt the implant-cement-bone interfaces. This determination was made at the time of surgery, and the surgeon was blinded to the synovial fluid analysis results until the study's conclusion.

### Patient Demographics

There were 50 patients consecutively enrolled in the study, and underwent perioperative aspiration at the time of revision TKA. Among the 50 patients, 26 (52%) were women, the mean age was 65 years (range, 43 to 85), and the mean body mass index was 31 (range, 20 to 45). Based on radiographic review and intraoperative confirmation, 21 patients had a titanium baseplate with a cobalt-chromium femoral component, while the remainder (29) had strictly cobalt-chromium components. Indications for revision included progressive pain (with or without radiographic evidence of loosening) (23), infection (12), instability (nine), osteolysis (two), extensor mechanism malfunction (two), and patellar maltracking (two).

### Data Analyses

Patients were stratified by their cement and implant composition to identify the ion(s) most predictive of loosening. First, patients were compared based on their implant composition, creating two groups: (1) cobalt-chromium only; and (2) cobalt-chromium femur with titanium baseplate. Patients who had confirmed loosening of a titanium baseplate were directly compared to patients who had well-fixed titanium components. A similar analysis was also performed for patients who had loose cobalt chromium implants. Next, patients were stratified by the radiotracer (zirconium versus barium), most likely present as determined by their cement or synovial aspirate. Patients who had zirconium-based cement and confirmed component loosening were then compared to patients who had similar cement and well-fixed components. The same analysis was also performed for patients who had barium cement. Analysis between loose and well-fixed implants was performed using Mann-Whitney tests, given the nonparametric distribution of the ion concentrations, and alpha was set at 0.05. Receiver operating characteristic curve analysis was then performed to provide the area under the curve (AUC) associated with each ion individually when classifying loose versus well-fixed. The AUC is reported with the 95% confidence interval (CI) and associated *P*-value and represents the accuracy of the screening test. Statistical analysis was performed using Statistical Package for the Social Sciences version 29 (IBM Corp., released in 2023. IBM SPSS Statistics for Windows, Version 29.0.2.0 (Armonk, New York: IBM Corp.).

### Microwave Digestion of Samples

In preparation for mass spectrometry and concordance with our laboratory’s protocols, acid digestion of synovial fluid samples was performed on the Milestone ETHOS UP Microwave Digestion System (Milestone Inc, Shelton, Connecticut). A volume of one mL of fluid was digested in a solution of nine mL of nitric acid (Fisher Chemical, trace metal grade [Fisher Scientific, Waltham, Massachusetts]) and one mL of hydrogen peroxide (Fisher Chemical, certified ACS grade). The microwave unit ran at a power of 1,800 watts and a temperature of 190°C. The digestion time was 30 minutes. To decrease the acid concentration of the digestate to less than 5% by volume before analysis on the mass spectrometer, the sample was diluted 20:1 with ultrapure water.

### Analyses of Samples

Inductively coupled plasma-mass spectrometry analysis of aqueous samples was conducted on a Perkin Elmer model NexION 2000B (PerkinElmer Corporation, Waltham, MA) in standard mode. The 7-point standard curves were used for an analytical range between 0.1 μg/L and 100 μg/L of a certified standard (AccuStandard, Inc, New Haven, Connecticut). Analytical blanks and analytical check standards at approximately 0.5 μg/L were run every 10 to 20 samples and required to be within 15% of the specified value, and the blanks were below method detection limits. Subsequently, 50 μL of a 10 mg/L internal standard (AccuStandard, Inc) consisting of lithium, yttrium, and indium to cover the full mass range of isotopes was added to 10 mL of sample aliquot before analysis. Instrument settings include 1.5 kW radio frequency power, 15 L/min argon plasma flow, 0.9 L/min nebulizer flow, and one second integration time for three replicates. Detection of metal ions was considered if the sample exceeded the lowest calibration standard, and the concentration in parts per billion (ppb) was recorded to compare relative concentrations.

## Results

**Figure 1.**
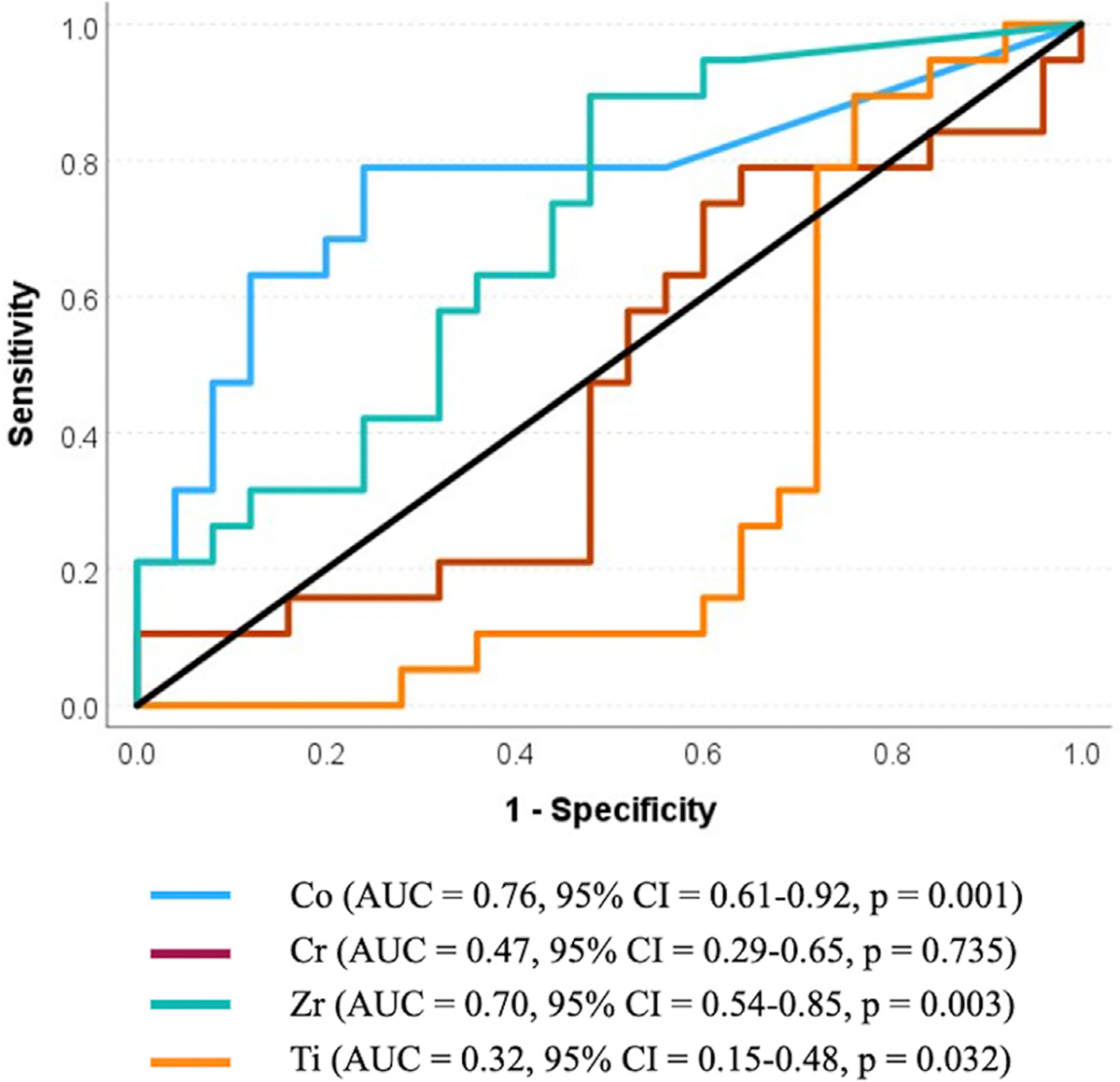
ROC curve for cobalt-chromium implants. ROC, receiver operating characteristic; AUC, area under the curve; Co, cobalt, Cr, chromium, Zr, zirconium, Ti, titanium.

There were 25 (50%) patients who had intraoperatively confirmed component loosening. When examining patients who had a titanium tibial baseplate, there were no differences in titanium, barium, or zirconium concentrations between loose and well-fixed implants (Table 1). Additionally, patients who had titanium components had similar levels of titanium synovial concentrations compared to those who did not have titanium implants (median: 208 versus 369 ppb, *P* = 0.2). Relative to patients who have well-fixed components, patients who have confirmed component loosening of cobalt-chromium implants had significantly elevated levels of zirconium (median levels: 17 versus four ppb, *P* = 0.03) and cobalt (median levels: 16 versus two ppb, *P* < 0.002) (Table 2). Cobalt had the strongest predictive power of loosening, with an AUC of 0.76 (95% CI = 0.61 to 0.92, *P* = 0.001), while zirconium also demonstrated good predictive power (AUC = 0.7, 95% CI = 0.54 to 0.85, *P* = 0.003) (Figure 1).

**Figure 2.**
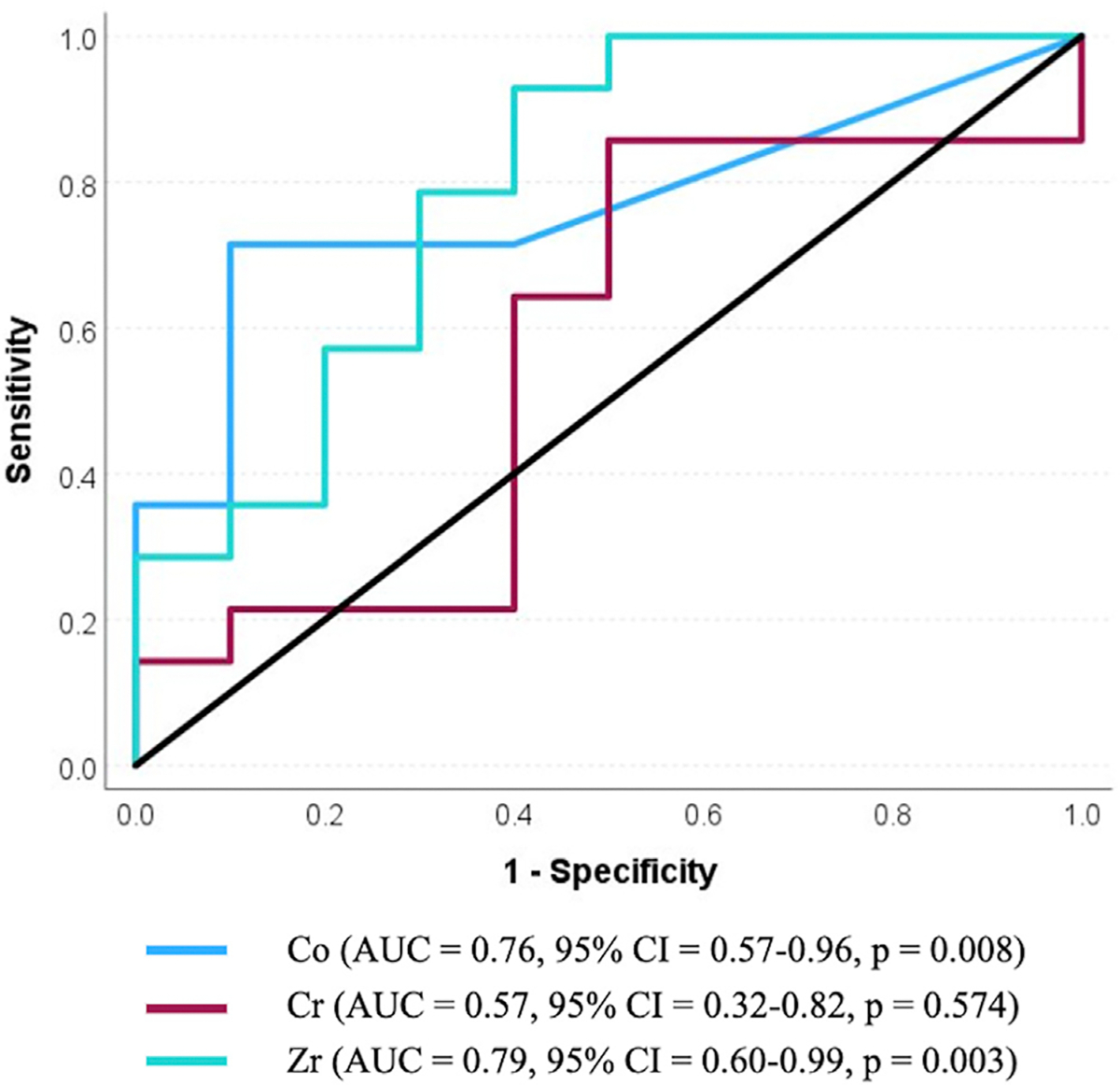
ROC curve for zirconium cement. ROC, receiver operating characteristic; AUC, area under the curve; Co, cobalt; Cr, chromium; Zr, zirconium.

Next, patients were evaluated based on the radiotracer most likely present in the cement during their index procedure. Patients who had confirmed or suspected zirconium-based cement, which had confirmed intraoperative component loosening, had elevated levels of both zirconium (median: 27 versus 6, *P* = 0.02) and cobalt (median: 22 versus 4, *P* = 0.031) compared to the well-fixed cohort (Table 3). These ions were also the most predictive synovial metal ion levels for diagnosing component loosening: cobalt (AUC = 0.76 (95% CI = 0.57 to 0.96), *P* = 0.008) and zirconium (AUC = 0.79 (95% CI = 0.6 to 0.99); *P* = 0.003) (Figure 2). Patients who had confirmed or suspected barium cement had no differences in barium synovial concentrations between loose and well-fixed components (median: 26 versus 22 ppb, *P* = 0.57), but did have increased levels of cobalt (median: 18 versus 2 ppb, *P* = 0.001) (Table 4).

## Discussion

Aseptic loosening remains a contemporary issue leading to clinical ambiguity [1–3,14]. In this study, we hypothesized that abrasive wear from loose TKA components would result in the release of metal ions, particularly zirconium and barium, into the synovial fluid. In a consecutive series of 50 patients undergoing revision TKA of primary components, we found that patients who had intraoperatively confirmed component loosening exhibited significantly elevated levels of zirconium and cobalt in their synovial fluid compared to those who had well-fixed implants. Specifically, the median synovial zirconium concentration was 27 ppb in patients who had loosening, compared to six ppb in those who did not (*P* = 0.016). Cobalt levels were also notably higher in the loosening group (16 versus 1.5 ppb, *P* = 0.002). There were, however, no significant differences in the levels of barium, chromium, or titanium between the two groups. These findings suggest that zirconium and cobalt levels could potentially serve as biomarkers for aseptic loosening in TKA.

Serial radiographs demonstrating implant migration are generally accepted as a diagnostic tool of aseptic component loosening. However, the best method to make this diagnosis in the absence of frank implant migration remains a point of debate. Some surgeons have advocated using the Knee Society Radiographic Evaluation System zones to evaluate implant loosening, which relies on detecting radiolucencies in zones underneath the implant [15]. However, in cases of debonding, radiolucent lines may not be present. Furthermore, major radiolucencies can be present without clinically relevant loosening, requiring revision [16,17]. Other imaging modalities, such as bone scans, CT scans, and single photon emission computed tomography scans, have been suggested as alternatives. Still, these also come with limitations, including difficulty in interpretation and a lack of specificity [9–11]. Given these challenges, there is a clear need for a non-invasive or minimally invasive test that can improve diagnostic accuracy and guide intervention.

In this study, 25 out of 50 patients had confirmed component loosening during surgery. For patients who had titanium tibial baseplates, there were no significant differences in synovial metal ion concentrations between loose and well-fixed implants. However, patients who had cobalt-chromium implants and confirmed loosening showed significantly elevated levels of zirconium and cobalt compared to those who had well-fixed components. Cobalt emerged as the most predictive marker for loosening, followed closely by zirconium. Among patients who had zirconium-based cement, those who had confirmed loosening had higher levels of both zirconium and cobalt, again suggesting their potential as reliable diagnostic markers. The combination of zirconium cement and cobalt-chromium implants provided the highest diagnostic accuracy, with 79% sensitivity and 100% specificity, indicating that synovial metal ion analysis could serve as a valuable, minimally invasive screening test for detecting aseptic loosening in TKA, potentially leading to earlier and more accurate diagnoses.

In contrast, barium concentrations did not differ significantly between loose and well-fixed implants. Although elevated levels of zirconium and cobalt were associated with loosening, the lack of significant differences in barium, chromium, and titanium concentrations suggests potential limitations in utilizing those markers for loosening.

Multiple factors could affect synovial ion testing for the purpose of identifying loose TKA implants. The variability in radiotracers and the potential inclusion of antibiotic bone cement, along with potential ion contamination from sources other than the implants [18], complicate the ability to reliably assess the role of these markers in diagnosing loosening. Differences in solubility and clearance rates among the metal ions and radiotracers could explain the differential findings of various ion concentrations in patients who have loosening. This variability may explain why cobalt, but not chromium, showed significant differences in concentration between loose and well-fixed implants despite both being present in most implants. There was heterogeneity in revision indication, which, if our proposed mechanism is true, may also influence the rate and nature of ion release and affect our results. Further studies are needed to understand the specific conditions under which these ions might serve as reliable biomarkers for aseptic loosening.

Although this study introduces a promising technique for identifying aseptic TKA failure, several potential limitations should be acknowledged. The potential for false positives or negatives in our screening test remains a major concern, particularly given the variability in ion concentrations and the unknown timing of ion release following implant loosening. While we showed the potential to detect aseptic loosening in cobalt chromium implants, our results are limited to these constructs, as our results were not predictive for titanium implants. Asymptomatic patients who had well-functioning prostheses were also not included, and the true baseline metal ion level following TKA is yet to be elucidated. Additionally, the radiotracer used in the initial surgery was not always known before aspiration, leading to instances where multiple ions, such as barium and zirconium, were present in the synovial fluid, complicating the analysis. Also, the relatively small sample size limits the generalizability of our results, and further studies with larger cohorts are necessary to identify diagnostic thresholds and better understand the baseline ion levels in patients who have well-fixed components. Addressing these limitations will be crucial for refining this screening technique and improving its clinical applicability. Furthermore, increasing the sample size will allow subgroup analysis, including a more critical evaluation of tibial versus femoral loosening and a greater variety of implant designs.

## Conclusions

In conclusion, our study highlights the potential of using synovial fluid metal ion concentrations, particularly zirconium and cobalt, as biomarkers for detecting loosening in TKA. Despite some limitations, including variability in ion solubility, clearance, and potential contamination from external sources, our findings suggest that elevated levels of these ions, especially in patients who had cobalt-chromium implants and zirconium-based cement, could serve as a useful screening tool for identifying implant loosening. The high specificity observed in our study underscores the potential clinical value of this approach in improving diagnostic accuracy and guiding timely intervention. However, further research with larger patient cohorts and optimized methodologies is needed to validate these findings and fully establish the sensitivity and specificity of this novel diagnostic test. Ultimately, with continued refinement, this screening technique could lead to earlier and more accurate detection of aseptic loosening, improving patient outcomes and reducing the need for unnecessary revisions.

## Supplementary Material

1

2

3

4

5

6

7

## Figures and Tables

**Table 1:** Ion Concentrations in Patients Who had Titanium Implants.

Metal Ion	Intraoperative Implant Evaluation^a,b^	Mean (ppb)	Median (ppb)	*P*-Value

Barium	Well-fixed	27	21	0.21
	Loose	328	22	
Zirconium	Well-fixed	17	3	0.35
	Loose	152	29	
Titanium	Well-fixed	449	369	0.54
	Loose	5,300	208	

ppb, parts per billion.

a Well-fixed patients = all patients with at least one titanium implant that was well-fixed.

b Loose patient = only patients that experienced loosening of titanium implant.

**Table 2:** Ion Concentrations in Patients Who had Cobalt-Chrome Implants.

Metal Ion	Intraoperative Implant Evaluation^a,b^	Mean (ppb)	Median (ppb)	*P*-Value

Barium	Well-fixed	25	14	0.76
	Loose	109	11	
Zirconium	Well-fixed	16	4	0.03
	Loose	74	17	
Chromium	Well-fixed	173	112	0.73
	Loose	488	110	
Cobalt	Well-fixed	5	2	<0.002
	Loose	152	16	

ppb, parts per billion.

a Well-fixed patients = Patients only had CoCr implants, and both were well-fixed.

b Loose patient = Loosening of either/both CoCr component(s).

**Table 3:** Ion Concentrations in Patients Who had Zirconium^a^-Based Cement.

Metal Ion	Intraoperative Implant Evaluation	Mean (ppb)	Median (ppb)	*P*-Value

Zirconium	Well-fixed	16	6	0.02
	Loose	144	27	
Titanium	Well-fixed	347	319	0.40
	Loose	455	264	
Chromium	Well-fixed	101	91	0.59
	Loose	210	113	
Cobalt	Well-fixed	4	4	0.031
	Loose	22	22	

ppb, parts per billion.

a Patients included if the cement or synovial fluid was indicative of Zirconium or indistinguishable between Ba and Zr.

**Table 4:** Ion Concentrations in Patients Who had Barium^a^-Based Cement.

Metal Ion	Intraoperative Implant Evaluation	Mean (ppb)	Median (ppb)	*P*-Value

Barium	Well-fixed	33	26	0.57
	Loose	216	22	
Titanium	Well-fixed	454	349	0.20
	Loose	3,013	273	
Chromium	Well-fixed	200	120	0.92
	Loose	642	137	
Cobalt	Well-fixed	6	2	0.001
	Loose	267	18	

ppb, parts per billion.

a Patients included if the cement or synovial fluid was indicative of barium or indistinguishable between Ba and Zr.
