# Supplementary material for: The AAHKS Surgical Techniques and Technologies Award: Synovial Fluid Metal Ion Levels as a Biomarker for Aseptic Loosening Following Cemented Total Knee Arthroplasty: A Prospective Study
Source: J Arthroplasty. Author manuscript; Available in PMC 2026 Jul 8. (PMC13345676; doi:10.1016/j.arth.2025.04.067)
Supplement: 5 [file NIHMS2176668-supplement-5.docx]

# INDIVIDUAL CONFLICT OF INTEREST STATEMENT

***American Association of Hip and Knee Surgeons***

(Adopted from the American Academy of Orthopaedic Surgeons disclosure statement)

The following form **must be filled out completely and submitted by each author (example, 6 authors, 6 forms).**

**All items require a response. If there is no relevant disclosure for a given item, enter "*None*.”**

**Synovial Fluid Metal Ion Levels as a Biomarker for Aseptic Loosening Following Cemented Total Knee Arthroplasty: A Prospective Study**

1. Royalties from a company or supplier (The following conflicts were disclosed)

None

2. Speakers bureau/paid presentations for a company or supplier (The following conflicts were disclosed)

None

3A. Paid employee for a company or supplier (The following conflicts were disclosed)

None

3B. Paid consultant for a company or supplier (The following conflicts were disclosed)

DePuy

3C. Unpaid consultants for a company or supplier (The following conflicts were disclosed)

None

4. Stock or stock options in a company or supplier (The following conflicts were disclosed)

None

5. Research support from a company or supplier as a Principal Investigator (The following conflicts were disclosed)

None

6. Other financial or material support from a company or supplier (The following conflicts were disclosed)

None

7. Royalties, financial or material support from publishers (The following conflicts were disclosed)

None

8. Medical/Orthopaedic publications editorial/governing board (The following conflicts were disclosed)

None

9. Board member/committee appointments for a society (The following conflicts were disclosed)

None

**Each author must sign AND print or type his/her name, date and submit a separate form**

In addition, one BLINDED Conflict of Interest form (no author names used) should be submitted per manuscript with all author disclosures.

Ryan Martin Ryan Martin 12/13/24

Author Name (Print or Type) Author Signature Date
